# Trajectories of loneliness in later life – Evidence from a 10-year English panel study

**DOI:** 10.1016/j.socscimed.2025.117703

**Published:** 2025-01-16

**Authors:** Giorgio Di Gessa, Valeria Bordone, Bruno Arpino

**Affiliations:** a Department of Epidemiology and Public Health, University College London, 1-19 Torrington Place, London, WC1E 7HB, UK; b Department of Sociology, University of Vienna, Austria; c Department of Statistical Science, University of Padua, Italy

**Keywords:** Loneliness, Social relationships, Social networks, Resources, Quality, Quantity, Support, Closeness, Friends, Extended family, Partner, Longitudinal, Lifecourse, ELSA

## Abstract

The prevalence of loneliness is higher among older people; however, few studies have examined its longitudinal patterns and associated risk factors, particularly social network resources. Using data from six waves of the English Longitudinal Study of Ageing (2008/09 to 2018/19, N = 4740) and group-based trajectory modelling (GBTM), we identified five groups of loneliness trajectories: three with stable levels (37% “stable low”; 26% “stable medium”; 9% “stable high”) and two with time-varying scores of loneliness (8% “increasing”; 20% “decreasing”). Multinomial regression models and GBTM were used to examine baseline and time-varying factors associated with these trajectories. We created composite measures to capture the presence and key facets of social network resources (including size, support, closeness, and frequency of contact) for four different social relationship domains (partner, children, immediate family, and friends). Our results show that, among those with family or friends, older people with higher social network resources and those who maintained or improved them over time reported lower levels of loneliness. Findings also suggest that all social relationship domains contributed to feelings of loneliness in later life. Also, we find that respondents in poor health and depressed, as well as those whose health deteriorated over time, were more likely to have stable high or increasing levels of loneliness. The study highlights the need to investigate loneliness from a life course perspective, account for the complexity of social relationships in later life beyond their mere presence or absence, and include their quality and quantity as well as multiple domains of family and intergenerational relationships.

## 1. Introduction

Loneliness is increasingly recognised as a public health concern, particularly at older ages. Loneliness is defined as a negative feeling that arises from both quantitative and qualitative discrepancies in individuals’ desired and actual social interactions and emotional support derived from social contacts and networks (Perlman and Peplau, 1981). A recent meta-analysis of the prevalence of loneliness across 113 countries shows that loneliness is a common experience worldwide, although its prevalence is heterogenous across countries (Surkalim et al., 2022). Moreover, while all age groups experience loneliness, a generally higher prevalence is observed among older adults in most countries (Barreto et al., 2021; Dykstra, 2009; Hawkley et al., 2022; Surkalim et al., 2022). Empirical evidence links loneliness to adverse health outcomes, such as cardiovascular diseases (Leigh-Hunt et al., 2017; Nicole et al., 2016), dementia and poorer cognitive function (Lara et al., 2019), as well as poorer wellbeing and mental health (Leigh-Hunt et al., 2017; Wang et al., 2018). Its association with increased risk of mortality (Holt-Lunstad et al., 2015; Rico-Uribe et al., 2018) is comparable to well-known risk factors such as smoking or lack of exercise (Office of the Surgeon, 2023).

Over the last few decades of scientific research on loneliness, several academic studies have investigated risk factors for loneliness in adult and later life, with recent meta-analyses and (systematic) reviews (Barjaková et al., 2023; Dahlberg et al., 2022; Morrish and Medina-Lara, 2021) summarising these findings whilst highlighting areas overlooked by previous literature on the topic. In particular, only recently has a growing body of research investigated loneliness using longitudinal data. However, many of these longitudinal studies are not focused on loneliness trajectories per se and their risk factors but treat them as predictors of subsequent physical or mental health and well-being. Moreover, although there is a general consensus on the importance of social networks, few studies have simultaneously considered the availability of partners, family, friends, and children, as well as different aspects of these personal relationships (such as size, quality, and frequency of contact). These omissions are critical given the complexity of family and intergenerational relationships in later life (Carr and Utz, 2020).

It is often hypothesised that the experience of loneliness can change across one’s life span depending upon an individual’s circumstances and perceptions: as people age, they may gain or lose partners, friends, and social contacts; increase or reduce opportunities to socialise; or even experience changes in the quality of their social relationships that might relate to their loneliness. As longitudinal data became available, initial studies have examined changes in loneliness between only two time points (Cohen-Mansfield et al., 2009; Dahlberg et al., 2018; Dykstra et al., 2005; Newall et al., 2014; Nicolaisen and Thorsen, 2014; Nyqvist et al., 2023; Victor and Bowling, 2012). However, this approach fails to capture the underlying trajectory of change over time (Parsons and McCormick, 2024), and it can mask the complexity of trajectories, particularly when absolute changes depend on the initial levels of loneliness and “no change” might include respondents both with persistence and absence of loneliness. More recently, researchers have increasingly used multiple waves of longitudinal studies to investigate the dynamic nature of loneliness (Aartsen and Jylhä, 2011; Gao et al., 2024; Huang et al., 2024; Li et al., 2023; Tiilikainen et al., 2024; Wenger and Burholt, 2004; Yang, 2018). However, most of these studies are interested in how loneliness trajectories predict physical or mental health and well-being. For instance, using data from four waves of the China Health and Retirement Longitudinal Survey, Huang et al. (2024) identified three loneliness trajectories and then investigated their associations with cardiovascular diseases, whereas Li et al. (2023), using data from the English Longitudinal Study of Ageing (ELSA), found five distinctive loneliness trajectories and used them to explore their association with dementia. Only a few of these longitudinal studies have examined the factors associated with different loneliness trajectories. Using Finnish data from a 10-year follow-up survey of older adults, Tiilikainen et al. (2024) found that the three relatively stable trajectories of loneliness identified in their study were associated with adverse circumstances in childhood and youth. Using five waves of ELSA, Yang (2018) also investigated sociodemographic and health characteristics associated with loneliness over time, although they did not allow for different loneliness trajectories. Therefore, this paper’s first major contribution is to describe trajectories of loneliness in later life and identify associated risk factors, including time-varying characteristics that might change as people age.

The concept of loneliness inherently involves considerations about also meaningful social connections and relationships. Although sometimes conflated with social isolation (Mansfield et al., 2023), loneliness may arise from not having family, partners, and friends as well as from the perception that these relationships are not satisfying or fulfilling enough (Aartsen and Jylhä, 2011). Although a large body of research has shown that having a partner, children, or friends is generally associated with lower levels of loneliness (Arpino et al., 2022; Dykstra et al., 2005; Nyqvist et al., 2023; Penning et al., 2022; Yang, 2018), scholars are increasingly also interested in relationships’ subjective quality, closeness, and frequency of contact (Shiovitz-Ezra and Leitsch, 2010). Previous studies have shown, for instance, that, although having a partner is important, not all partnered people report similar levels of loneliness, with those in supportive and close relationships reporting lower feelings of loneliness (Margelisch et al., 2017; Warner and Adams, 2015). Similarly, previous studies have highlighted how loneliness relates to the frequency of contact, closeness, and quality of relationships with children as well as extended family and friends (Dahlberg et al., 2018; de Jong Gierveld et al., 2009; Hawkley and Kocherginsky, 2018). To date, however, most studies not only have paid little attention to family members beyond partners and children (despite their potential contribution to feelings of loneliness) but have also focused on the mere presence or absence of social networks, overlooking important aspects of those relationships (such as size, perceived social support, closeness, and frequency of contact). Therefore, this study’s second novel contribution to the field is to consider a wide range of important domains of social relationships (including extended family and friends) and account for their presence or absence as well as the quality of such relationships.

Overall, this study aims to provide valuable additional insights into the dynamic nature of loneliness as people age and to examine factors associated with loneliness trajectories, particularly social relationships. First, we describe 10-year loneliness trajectories among older people in England. Second, we analyse how changing individual demographic, socioeconomic, health, and social characteristics relate to loneliness trajectories. Our analyses will consider the widest definition of social relationships (partners, children, immediate family, and friends) and a range of social network characteristics (such as size, perceived social support, closeness, and frequency of contact).

## 2. Materials and methods

### 2.1. Study design and participants

Data were obtained from the English Longitudinal Study of Ageing (ELSA), an interdisciplinary ongoing cohort study of older adults aged 50 years and older living in private households in England (Banks et al., 2021). The study sample is periodically refreshed with new participants to ensure the study remains representative of those aged 50 and over. Each sample is drawn from households that had previously responded to the Health Survey for England (HSE). The HSE is an annual cross-sectional survey that uses a clustered stratified probability sampling technique and is designed to monitor the general population’s health. The study started in 2002, with an initial individual response rate of 67%. Data are collected biennially using face-to-face personal interviews and self-completion questionnaires. Details of the survey’s sampling frame, methodology, and questionnaires can be found at www.elsa-project.ac.uk. ELSA was approved by the London Multicentre Research Ethics Committee (MREC/01/2/91). Informed consent was obtained from all participants. All data are available through the UK Data Service (SN 5050).

Our sample consisted of non-proxy participants who had been interviewed in both wave 4 (2008/9) and wave 9 (2018/9) and had filled in the self-completion questionnaire where the main variables of interest were collected. Of the initial 8988 non-proxy respondents with available data at wave 4, 4740 participants (53%) returned the self-completion questionnaire at wave 9. Among the final analytical sample, 93% of respondents were present in all six waves under study, with 80% completing the self-completion questionnaire at all waves.

### 2.2. Main measurements of interest

#### 2.2.1. Outcome

Loneliness was measured in the self-completion questionnaire using the short revised version of the University of California, Los Angeles (RUCLA) Loneliness Scale (Hughes et al., 2004), designed to measure loneliness without directly mentioning the word “loneliness”. The R-UCLA loneliness scale includes three questions: “How often do you feel you lack companionship?”, “How often do you feel left out?” and “How often do you feel isolated from others?”. Responses were scored from 0 (*hardly ever or never*) to 2 (*often*), with total scores ranging from 0 to 6 and higher values indicating greater loneliness. The scale has high validity and is internally consistent (Cronbach’s alpha = 0.82). Although previous studies have often considered values ≥ 3 (i.e. values in the top quintile of the distribution) to represent high loneliness (Davies et al., 2021; Gale et al., 2017; Steptoe et al., 2013), there is no specified and validated threshold score for the R-UCLA loneliness scale. Therefore, we considered values as zero-inflated count data because of the scale’s positively skewed distribution (mean = 1.1; median = mode = 0).

#### 2.2.2. Time-varying covariates

In line with the risk factors for loneliness examined in the most recent reviews (Dahlberg et al., 2022), we accounted for a wide range of socioeconomic, social network resources, and health-related time--varying covariates. This allows us to identify the effects of these dynamic variables on changes in loneliness scores. The time-varying covariates were contemporaneous with loneliness at each wave.

As time-varying socioeconomic factors, we included employment status (with respondents being classified as in paid work if they described their current economic situation as employed or self-employed) and voluntary work in the previous month.

Health-related factors included self-perceived health, physical disability, depression, as well as vision and hearing difficulties. Self-rated health (SRH) was measured on a five-point ordinal scale (excellent, very good, good, fair, or poor). The five SRH items were dichotomised into “fair or poor” versus better health. Physical disability was assessed using limitations in activities of daily living (ADL), such as getting out of bed and walking across a room, and instrumental ADL, such as shopping for groceries and preparing a hot meal. Participants who reported limitations with one or more activities were defined as having physical impairments. Mental health was measured using the 8-item short version of the Centre for Epidemiologic Studies Depression (CES-D) scale, which has been validated as a reliable measure of depressive symptomatology in older adults (Karim et al., 2015). The scale includes eight binary (no/yes) questions about whether respondents experienced any depressive symptoms, such as feeling sad or having restless sleep, the week before the interview. We classified respondents who reported four or more depressive symptoms on the CES-D scale as having elevated depressive symptoms. Furthermore, respondents were asked to rate their eyesight and hearing, and answers ranged from excellent to poor. People were defined as having difficulties with vision or hearing if they rated their vision or hearing as fair or poor. Finally, we considered sedentary behaviour, which is defined as hardly ever doing any physical activity or engaging only in mild leisure-time activities.

Among indicators of social network resources, we considered several questions that accounted not only for the presence of partners, children, immediate family, and friends but also for their size, support, and frequency of contact. For each relationship domain (with partner, children, immediate family, and friends), respondents were asked to rate their perception of social *support* (with six statements including how much they “can rely on them” or “understand the way they feel” and response choices ranging from “not at all” to “a lot”). Respondents with available relationships were also asked to report how close their relationship was with their partner (very close vs less) and how many children, family members, and friends they have a close relationship with (with options ranging from zero to 5 or more). ELSA participants were also asked to report, on average, how often they meet up, speak on the phone, or write/email with children, family, and friends (with answers ranging from “three or more times a week” to “less than once a year or never”). Similarly to Litwin and Stoeckel (2016), we created four scales (one for partners, one for children, one for other family members, and one for friends) that incorporated all of these characteristics into composite measures to capture key facets of social networks (*size*, *support, closeness*, and *frequency of contact*) within one indicator. After adding all these values for each domain, we used tertiles. Therefore, we distinguished between those who had no partner/family/friends and –among those who did –between respondents with social network resources ranging from “low” to “high”.

#### 2.2.3. Time-stable covariates

Although age, education, wealth, and gender were assessed at each wave, we considered these covariates time-constant as they showed little or systematic variability over time. Age at baseline was modelled as a categorical variable, distinguishing those aged 50–59, 60–69, and 70+. The educational level was re-coded as low (below secondary), middle, and high (university or above), following the International Standard Classification of Education (http://www.uis.unesco.org/). Wealth was equal to the total net non-pension non-housing wealth, and respondents were categorised into tertiles.

### 2.3. Statistical analysis

We conducted two steps of analysis. First, we applied group-based trajectory modelling (Nagin and Odgers, 2010) to identify distinctive trajectory patterns of loneliness. This method takes into account the dependency of observations and assumes a mixture of subpopulations with different individual trajectories within the target population and identifies distinctive groups within which individuals share similar developmental trajectories (Herle et al., 2020; Nguena Nguefack et al., 2020). The model can capture linear and nonlinear trajectories by introducing additional quadratic or cubic growth parameters. Missing data were handled using full information maximum likelihood estimation. For each subject, the model provides the probability of belonging to each identified trajectory group and assigns the subject to the trajectory group based on the highest probability. To determine the number of trajectory groups within our sample, we tested 2–7 unconditional group-based trajectory models using the six waves of data, with the optimal number of groups selected using a wide range of criteria, including the Akaike Information Criterion (AIC), the Bayesian Information Criterion (BIC), and its sample size-corrected version (c-BIC). For each of these, lower scores indicate (relatively) better fitting models. Moreover, we considered the overall average posterior probabilities of group membership as a measure of classification quality (entropy index, with values approaching 1.0 indicating a favourable classification); the average posterior probability assignment for each group (>70%); the odds of correct classification (>5.0); group size (and the avoidance of groups smaller than 5% that may lead to lack of reproducibility of the results); the usefulness of the number of groups in terms of the similarities/differences in their trajectory shapes; and the interpretability of the distinctive trajectories (Nagin and Odgers, 2010; Nguena Nguefack et al., 2020). After determining the optimal number of trajectory groups, we established the optimal shape of the trajectories, allowing them to be modelled as linear, quadratic, or cubic. Higher order growth parameters (quadratic to quintic) were dropped if not statistically significant (p < 0.05).

Second, once the trajectories were identified, we adopted two strategies to examine factors associated with these trajectories of loneliness. In an initial model, we used a multinomial regression model and considered all covariates at baseline (i.e. Wave 4) to identify factors associated with loneliness trajectory groups. To ease the interpretation of the results, we present average marginal effects (AMEs) that, due to the categorical nature of our outcomes and covariates, are to be interpreted as the average change in the probability of the outcome across the categories of the covariates being examined. AMEs are summary measures that estimate a predictor variable’s partial effect (in percentage points) on the outcome. In a second model, we used group-based trajectory modelling to fully exploit the longitudinal nature of the data and identify time-constant and time-varying covariates associated with scores of loneliness within each loneliness trajectory. Incorporating time-varying predictors in the model allows for a trajectory to depend on additional variables beyond time. The effects of time-varying covariates are estimated for each given trajectory group, allowing them to differ among trajectory groups. Estimates for time-varying covariates represent, for each trajectory, the average increase/decrease in loneliness score per unit change in the exposure. Data management, trajectories, and statistical analyses were performed using Stata/MP 18.0 (and the *traj* plugin).

## 3. Results

### 3.1. Loneliness trajectories

Table 1 shows the average values of the R-UCLA loneliness scale across all six waves under study, including the percentages that report values equal to zero and more than 3. Overall, the average loneliness score is stable over time at around one, with the majority of respondents (about 56%) reporting a score of zero and ~16/18% a R-UCLA ≥3 at each wave.

**Fig. 1.**
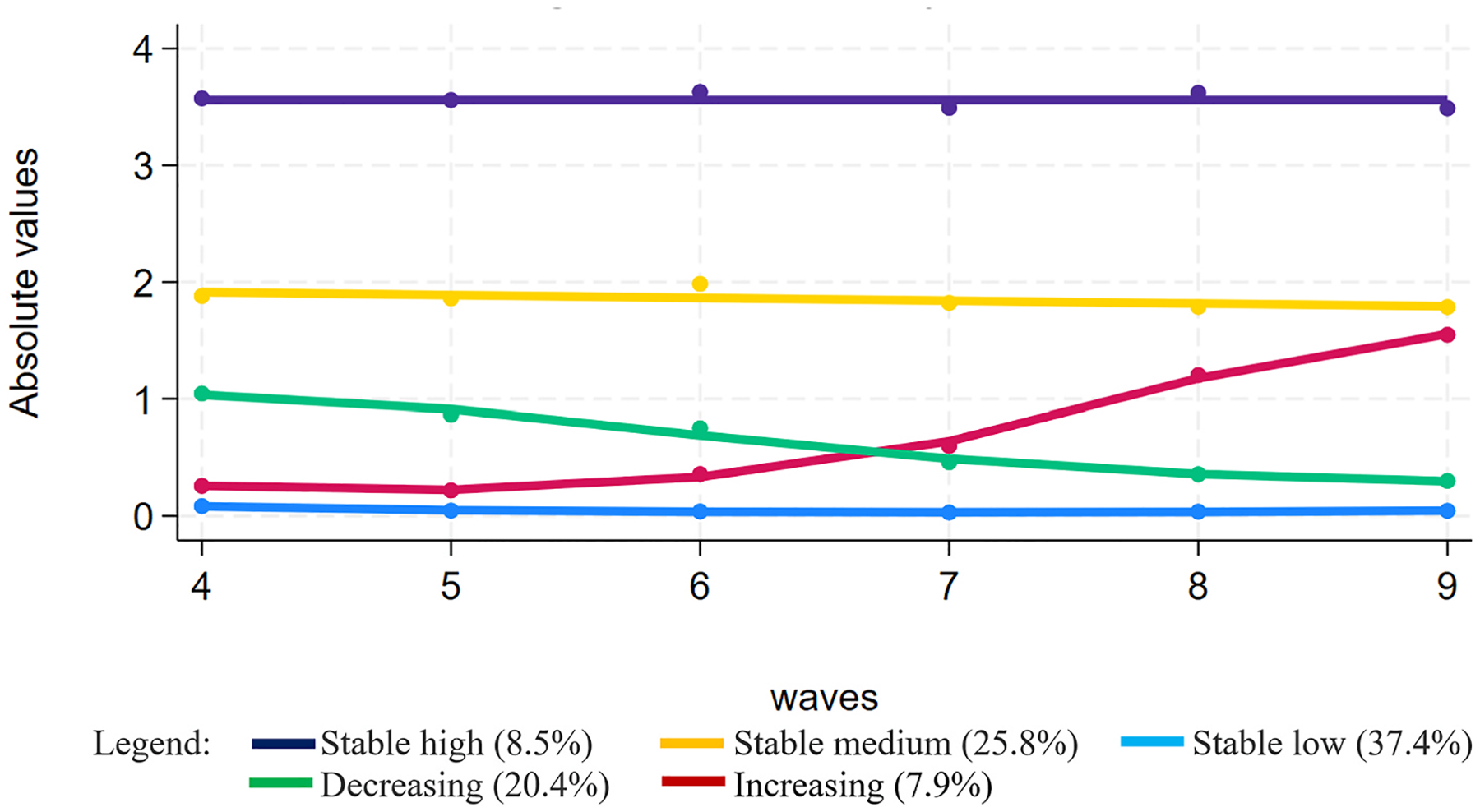
Loneliness trajectories. Trajectories obtained with an unconditional model that accounts only for time (expressed as the wave of the study). The dots represent the observed average scores, whereas the lines represent the predicted averages over time. Source: English Longitudinal Study of Ageing (ELSA) waves 4 (2008/09) – 9 (2018/19).

To summarise the development of loneliness over time and determine the optimal number of trajectory groups, a series of group-based trajectory models were fitted (with a specification of up to seven trajectory groups). Based on the abovementioned criteria (see Supplementary Tables 1–3), we identified five as the number of trajectory groups that best fit the data. The groups identified as a function of time (expressed by the wave of the study) are shown in Fig. 1. Three groups represent respondents with stable scores of R-UCLA loneliness, with 37% reporting low loneliness (“stable low”), 26% medium (“stable medium”), and 9% high loneliness (“stable high”) throughout. The remaining two classes show time-varying scores of loneliness, with 8% of respondents progressively increasing their average scores of loneliness (“increasing”) and about 20% reporting progressively lower scores of loneliness (“decreasing”) over time. As specified in the appendix (Supplementary Table 4), the “increasing” group starts at around 0.16 average R-UCLA loneliness score (with 87% reporting no loneliness at all) and ends up with a value of 1.72. For the decreasing loneliness group, the average loneliness scores decrease by about 1 point in ten years (from 1.13 to 0.27).

### 3.2. Baseline characteristics of loneliness trajectory group membership

Table 2 summarises ELSA respondents’ baseline characteristics and shows the distributions of potential risk factors among the five loneliness trajectory groups. Overall, the percentage of women in the “stable high” and “stable medium” loneliness groups is higher than in the overall sample distribution. Also, among those in the “increasing” loneliness group, there is a higher percentage of respondents aged 70 and older than in other groups. Those in the “stable low” loneliness group tend to be socioeconomically better off (with a higher percentage of respondents in this group having medium-high levels of education, being in the top tertile wealth distribution, and being in paid work). At baseline, there were also stark health contrasts across the loneliness trajectories groups, with respondents in the “stable low” group least likely to report any of the health issues considered and those in the “stable high” reporting the poorest mental and physical health and highest levels of physical inactivity.

When social relationships are considered, Table 2 shows that those in the “stable high” loneliness have the highest percentages of respondents with no partner, no children, and no friends at baseline. However, among those with a partner, children, immediate family, or friends, the relationship quality and the frequency of contact differ across different loneliness trajectories. Among respondents in the “low stable” loneliness group, we observe higher levels of social network resources with partners, children, immediate family, and friends (whereas those in the “stable high” loneliness report more frequently low levels of social network resources in all relationship domains).

Table 3 shows results from the multinomial logistic regression analyses regarding the associations between “entry-level” baseline demographic, socioeconomic, social relationships, and health factors and groups of loneliness trajectories. Results are reported as average marginal effects (AMEs). Women were more likely to be in the “stable high” loneliness group than men, while those in their 60s and 70s were more likely to be in the “increasing” loneliness group and less likely to be in the “decreasing” group compared to those in their 50s. Wealthier respondents were 4.2 percentage points more likely to be in the “stable low” loneliness group and 2.5 percentage points less likely to be in the “increasing” group than those in the lowest wealth tertile. With regards to health, respondents in poor health at baseline were less likely to be in the “stable low” loneliness group and significantly more likely to be in the “stable medium” or “stable high” loneliness groups. Health status, however, was not associated with varying loneliness trajectories, except for depressed respondents at baseline who are 3.6 percentage points less likely to belong to the “increasing” loneliness group. Finally, social network resources from partners, children, immediate family, and friends were significantly associated with loneliness trajectories, with higher resources associated with the “stable low” loneliness group. For example, those with high resources from friends were 11.3 percentage points *more* likely to be classified in the “stable low” loneliness trajectory group and 4.8 percentage points *less* likely to be in the “stable medium” or “stable high” groups. Generally, with a few exceptions, there were no differences in loneliness trajectory groups between those with low social network resources and those without resources at all.

### 3.3. Time-varying predictors of trajectories of loneliness

Significant differences in time-varying health and social relationships were found among the loneliness groups (see Supplementary Table 5 for details). For example, “stable low” loneliness group members had the best health profiles, and high and stable social network resources. The “increasing” and “decreasing” loneliness groups started with similar health and social resources, but their trajectories diverged. For example, the “decreasing” group’s health and resources improved or did not deteriorate as quickly as those in the “increasing” loneliness group.

Table 4 shows the fully adjusted associations between time-varying covariates and the trajectory of loneliness within each group. Overall, poorer health was associated with higher levels of loneliness across all loneliness groups, with depressive symptoms associated with higher levels of loneliness (coefficients range from 1.542 for “stable low” to 0.305 for “stable high”). Higher social network resources were associated with lower loneliness across all trajectory groups. For example, reporting high resources from friends is associated with lower loneliness scores (from −0.979 for “stable low” to −0.147 for “stable high”). This pattern is observed at different strengths and levels for all social domains, suggesting that improving social network resources was associated with reducing loneliness scores. Moreover, no longer having a partner is associated with higher levels of loneliness in all trajectory groups. Similarly, lack of friends increases loneliness, though not significantly in the “stable low” group (almost all members of this group report having friends throughout the waves under study). Not having children or immediate family members is not significantly associated with changes in loneliness compared to those who report low resources from these domains of social relationships.

## 4. Discussion

This study shows the overall stability of loneliness in later life over ten years, although at different levels. Using data from six waves of ELSA, we found that 37% of older people in England had stable low levels of loneliness, about 9% reported high loneliness throughout the period of observation, with a quarter of respondents classified as having stable medium loneliness. These results align with a recent meta-analysis that indicates mean-level stability of loneliness across the lifespan (Mund et al., 2020), with findings described as similar to those observed for other personality traits. Previous longitudinal studies also reported relatively stable levels of loneliness; for example, Huang et al. (2024) reported that 42% of respondents in China had stable low loneliness, whereas 10% were classified as having relatively high loneliness scores during the 7-year follow-up period. Similarly, Tiilikainen et al. (2024) reported 36% and 14% of Finnish older adults with low and severe loneliness trajectories during the seven years of follow-up, respectively. Our study not only highlights the heterogeneity in the level of stability but also shows the presence of trajectory groups with opposite trajectories of loneliness. In our study, just over a quarter of the sample experienced trajectories of changes in loneliness, with 20% reporting a slight decrease in loneliness and 8% experiencing increasing levels of loneliness. Similar patterns were also observed by Li et al. (2023) in their 8-year analysis of loneliness trajectories, although the authors reported different prevalences of these groups (at around 12% each) in their study. Our analyses also suggest that it is mostly those in their 60s and 70s who were more likely to be in the “increasing” loneliness group – this chimes with recent findings from a coordinated analysis of nine longitudinal ageing studies that found an increase in loneliness in older adulthood (Graham et al., 2024).

Unlike many previous studies that focused solely on baseline predictors of loneliness over time, our study also considered several factors that likely fluctuate over time and interact in complex ways to shape dynamic loneliness. In particular, we considered relationship quality, which could impact social support availability and perceptions of loneliness (Shiovitz-Ezra and Leitsch, 2010), particularly when older people are more likely to prioritise emotionally meaningful bonds (Carstensen et al., 1999). Our analyses find that loneliness trajectories and changes over time highly depend on initial levels of and changes in social network resources. In particular, we found that not only is a lack of friends and family detrimental to loneliness, but also that having few relationships, low perceived social support, closeness, and infrequent contact are significant predictors of “stable high” and “increasing” loneliness. Although this aligns with cross-sectional studies (Aartsen and Jylhä, 2011; Dahlberg et al., 2018; de Jong Gierveld et al., 2009; Hawkley and Kocherginsky, 2018), our longitudinal analysis of the link between social network resources and perceived loneliness sheds further light on this complex relationship. In particular, we found that reducing social network resources is associated with higher loneliness over time, with losing partners and friends particularly detrimental to loneliness. However, positive shifts are as important, and those older people who maintain or increase contact, closeness, and support with partners, children, and friends are more likely to report lower loneliness scores (with a less clear and weaker role of immediate family members). For instance, among respondents in the decreasing loneliness group, we observe an overall improvement in social network resources. In contrast, among those experiencing increases in loneliness, there are substantial reductions in social network resources (as well as increases in widowhood and loss of friends).

Our analysis also shows that poorer health, as well as deteriorating health (and depression in particular), are strong predictors of the likelihood of increasing loneliness over time, regardless of the initial levels of loneliness. These findings are in line with most studies on loneliness that suggest strong associations between poor physical and mental health and loneliness in older adults (Barjaková et al., 2023; Dahlberg et al., 2022), although it is often difficult to rule out the bi-directionality of these relationships. Finally, our results suggest that the overall levels of loneliness experienced by each trajectory group relate to both patterns and the overall prevalence of social network resources and health. It is also important to acknowledge that these factors might cancel each other out if acting in different directions. Studies have shown, for example, that older people can change their coping mechanisms and reorganise their social relationships, adapting to events such as widowhood by increasing support and contact with their friends, children, or other relatives (Freak-Poli et al., 2022).

### 4.1. Strengths and limitations

Strengths of this study include using a representative sample of the general population of older people living in private households in England and using multiple waves of the study that cover a period of ten years. Also, we used the R-UCLA loneliness scale, often considered more reliable than direct questions on loneliness that might elicit socially desirable answers, potentially under-reporting loneliness (Victor et al., 2000). Furthermore, by using group trajectory modelling, we were able to identify homogenous groups of older people that share similar trajectories of loneliness while capturing more heterogeneity in their levels. Also, in this study, we used an extensive battery of repeated measures on social network resources. In particular, we could investigate the complex role of social relationships on loneliness in later life as ELSA collects information on size, closeness, perceived social support, and frequency of contact for four different domains of social relationships: partners, children, immediate family, and friends.

Despite these advantages, our study has some limitations. First, when studying loneliness, sample attrition matters as the loss to follow-up (mostly because of death but possibly also due to admission to care homes) might be higher among those who feel lonely (Holt-Lunstad et al., 2015; Rico-Uribe et al., 2018). We might thus overestimate “low” levels of loneliness and underestimate “stable high” levels, as well as the group with increasing levels of loneliness. Second, questions on loneliness and social network resources have no temporal framework, so it remains difficult to disentangle the bi-directionality of these links. Third, although we controlled for baseline age and gender, we did not investigate whether trajectories of loneliness (and their relationships with health and social network resources) differed by these characteristics. Although changes in loneliness might be attributed to individual experiences rather than age and sex, future studies could investigate this issue further. Similarly, our findings about levels and trajectories of loneliness might be country-specific. Future studies should investigate whether similar results are observed in other populations and settings, particularly given the considerable cross-national variation in loneliness in later life (Sheftel et al. 2024). Finally, given the design of ELSA, which samples only the over-50 population and excludes those in care homes, we could not evaluate loneliness trajectories across the full adult-age spectrum.

## 5. Conclusion

The results of this study indicate that social relationships and health play a crucial role in loneliness in later life. In particular, our analysis highlights the complexity of social network resources and their importance in shaping loneliness beyond the mere presence of partners, family, and friends. Although not having any networks is undoubtedly associated with a higher risk of persistent loneliness, having family and friends is not necessarily counterbalancing such a risk if the perceived quality of these relationships is not satisfactory. When analysing factors that shape loneliness over time in later life, it is therefore important to explore how changes in social relationships and health interrelate with loneliness trajectories, making sure that both quality and quantity, as well as several domains of these relationships, are considered to fully account for the complexity of family and intergenerational relationships in later life. If policymakers are interested in understanding who is most at risk of loneliness in later life and in designing targeted policy interventions and programmes that tackle loneliness, attention should be placed on the ability to maintain both health and adequate and supportive social relationships.

## Supplementary Material

Appendix A

## Figures and Tables

**Table 1:** Summary of longitudinal data on R-UCLA loneliness scores.

	N	Mean (SD)	R-UCLA = 0	R-UCLA ≥ 3
Wave 4	4,740	1.08 (1.45)	2,490 (52.5%)	850 (17.9%)
Wave 5	4,470	1.00 (1.40)	2,490 (55.7%)	768 (17.2%)
Wave 6	4,408	1.03 (1.42)	2,414 (54.8%)	779 (17.7%)
Wave 7	4,366	0.94 (1.37)	2,530 (57.9%)	690 (15.8%)
Wave 8	4,372	0.98 (1.41)	2,477 (56.7%)	718 (16.4%)
Wave 9	4,740	1.00 (1.42)	2,684 (56.6%)	836 (17.6%)

Source: ELSA waves 4–9.

**Table 2:** Baseline sample characteristics (%), by loneliness trajectories and overall.

	Group 1 *Stable low*	Class 2 *Increasing*	Class 3 *Decreasing*	Class 4 *Stable medium*	Class 5 *Stable high*	*Total*	*P value*
**Demographic and Socioeconomic characteristics**							
Female	51.8	52.1	54.3	61.5	69.4	56.3	<0.001
50–59	37.5	30.0	43.6	37.7	41.4	38.5	<0.001
60–69	44.2	42.0	38.2	38.6	38.8	40.9	
70+	18.3	28.0	18.2	23.7	20.2	20.6	
Mean age (SD)	62.4	64.4	62.0	63.2	62.2	62.6	<0.001
High education	25.8	23.8	22.0	21.6	13.9	22.8	<0.001
Lowest wealth tertile	26.1	33.8	32.4	37.4	54.6	33.4	<0.001
Highest wealth tertile	40.7	30.5	34.2	27.6	18.4	33.3	
In paid work	48.1	39.0	49.4	39.7	36.8	44.5	<0.001
Volunteered	19.8	17.1	17.2	18.9	12.9	18.2	0.019
**Health**							
Fair/poor SRH	10.3	16.8	16.0	21.4	39.9	17.4	<0.001
Disability	11.8	18.2	16.1	23.5	37.1	18.4	<0.001
Depressed	3.2	4.0	7.9	14.2	42.3	10.4	<0.001
Fair/Poor Vision or Hearing	18.9	23.8	22.5	23.9	32.1	22.4	<0.001
Sedentary Behaviour	6.5	9.9	8.5	9.9	18.2	9.0	<0.001
**Social Relationships**							
No **partner**	11.7	11.4	23.1	31.3	48.4	22.2	<0.001
Partner – Low resources*	16.6	26.3	39.0	52.5	73.3	33.3	<0.001
Partner – Medium resources *	49.8	53.2	44.8	36.4	21.8	44.5	
Partner – High resources *	33.6	20.5	16.2	11.1	4.8	22.2	
No **children**	12.9	9.7	13.1	14.7	16.7	13.5	0.035
Children – Low resources*	27.9	31.3	38.8	46.5	60.7	37.8	<0.001
Children – Medium resources *	39.3	39.1	36.7	34.1	24.9	36.3	
Children – High resources *	32.8	29.6	24.5	19.4	14.4	25.9	
No **immediate family**	6.9	4.9	5.0	7.3	6.0	6.4	0.122
Immediate Family – Low resources*	28.6	31.2	35.8	37.4	48.4	34.2	<0.001
Immediate Family – Medium resources *	31.0	30.9	30.6	29.1	32.1	30.5	
Immediate Family – High resources *	40.4	37.9	33.6	33.5	19.5	35.3	
No **friends**	2.1	3.5	3.7	4.4	8.6	3.7	<0.001
Friends – Low resources*	23.3	28.8	30.0	31.8	39.9	28.6	<0.001
Friends – Medium resources *	32.5	36.7	35.1	34.7	36.6	34.2	
Friends – High resources *	44.2	34.5	34.9	33.5	23.5	37.2	
**Respondents (%)**	**1,771 (37.4%)**	**374 (7.9%)**	**968 (20.4%)**	**1,225 (25.8%)**	**402 (8.5%)**	**4,740**	

Notes: P-values are obtained from chi-squared or ANOVA tests. Social network “resources” are obtained by combining size, perceived social support, closeness, and frequency of contact for each of the domains considered (partner, children, immediate family, friends);

* these percentages (and the corresponding p-value) are restricted to respondents with social relationships in each of the domains.

Source: English Longitudinal Study of Ageing (ELSA) waves 4 (2008/09) – 9 (2018/19).

**Table 3:** Average marginal effects for a multinomial logistic regression model for loneliness trajectories.

	Group 1 *Stable low*	Class 2 *Increasing*	Class 3 *Decreasing*	Class 4 *Stable medium*	Class 5 S*table high*
Female	−0.024 (0.014)	−0.005 (0.008)	−0.020 (0.013)	0.026 (0.014)	0.022 (0.008) **
Age: 60–69 (Ref: 50–59)	0.048 (0.016) **	0.023 (0.009) *	−0.041 (0.015) **	−0.023 (0.016)	−0.007 (0.009)
Age: 70+ (Ref: 50–59)	0.011 (0.022)	0.056 (0.015) ***	−0.045 (0.020) *	−0.002 (0.021)	−0.021 (0.011)
High Education	−0.000 (0.016)	0.013 (0.011)	−0.023 (0.014)	0.025 (0.016)	−0.015 (0.010)
Highest wealth tertile (Ref: Lowest)	0.042 (0.017) *	−0.025 (0.011) *	0.021 (0.016)	−0.019 (0.017)	−0.018 (0.010)
Middle wealth tertile (Ref: Lowest)	0.006 (0.017)	−0.011 (0.011)	0.008 (0.015)	0.008 (0.016)	−0.012 (0.009)
In paid work	0.021 (0.016)	−0.006 (0.010)	0.017 (0.015)	−0.029 (0.016)	−0.002 (0.009)
Volunteered	0.001 (0.017)	−0.009 (0.010)	−0.007 (0.016)	0.021 (0.017)	−0.006 (0.010)
Fair/poor SRH	−0.074 (0.021)***	0.009 (0.013)	0.009 (0.018)	0.035 (0.019)	0.021 (0.010) *
Disability	−0.058 (0.020) ***	−0.002 (0.011)	−0.007 (0.018)	0.041 (0.019) *	0.027 (0.010) **
Depressed	−0.153 (0.024) ***	−0.036 (0.013) **	0.019 (0.022)	0.076 (0.024) **	0.133 (0.016) ***
Fair/Poor Vision or Hearing	−0.013 (0.016)	0.001 (0.010)	−0.002 (0.015)	−0.003 (0.015)	0.016 (0.009)
Sedentary Behaviour	−0.011(0.026)	0.018 (0.016)	0.001 (0.023)	−0.035 (0.022)	0.005 (0.011)
No partner	−0.016(0.019)	−0.041 (0.010) ***	0.003 (0.019)	0.003 (0.021)	0.051 (0.014) ***
Partner – Low resources	*Ref*	*Ref*	*Ref*	*Ref*	*Ref*
Partner – Medium resources	0.227 (0.018) ***	0.028 (0.011) ***	−0.039 (0.016) **	−0.147 (0.017) ***	−0.068 (0.009) ***
Partner – High resources	0.376 (0.022) ***	0.007 (0.013)	−0.085 (0.018) ***	−0.215 (0.019) ***	−0.082 (0.010) ***
No children	0.052 (0.022) *	−0.002 (0.014)	−0.016 (0.019)	−0.034 (0.021)	−0.004 (0.012)
Children – Low resources	*Ref*	*Ref*	*Ref*	*Ref*	*Ref*
Children – Medium resources	0.065 (0.017) ***	0.010 (0.010)	0.004 (0.015)	−0.047 (0.016) **	−0.031 (0.009) ***
Children – High resources	0.082 (0.019) ***	0.018 (0.011)	0.002 (0.017)	−0.071 (0.018) ***	−0.031 (0.011) ***
No immediate family	0.056 (0.028)	−0.016 (0.015)	−0.047 (0.025)	0.031 (0.028)	−0.024 (0.016)
Immediate Family – Low resources	*Ref*	*Ref*	*Ref*	*Ref*	*Ref*
Immediate Family – Medium resources	0.049 (0.017) **	0.006 (0.010)	−0.010 (0.015)	−0.030 (0.016)	−0.016 (0.010)
Immediate Family – High resources	0.060 (0.017) ***	0.013 (0.010)	−0.015 (0.015)	−0.011 (0.016)	−0.046 (0.009) ***
No friends	−0.052 (0.037)	0.008 (0.024)	0.011 (0.035)	0.013 (0.037)	0.020 (0.022)
Friends – Low resources	*Ref*	*Ref*	*Ref*	*Ref*	*Ref*
Friends – Medium resources	0.052 (0.017) ***	0.008 (0.011)	−0.003 (0.015)	−0.035 (0.016) *	−0.022 (0.010) *
Friends – High resources	0.113 (0.017) ***	−0.008 (0.010)	−0.008 (0.016)	−0.048 (0.017) **	−0.048 (0.010) ***

Notes: These findings are reported as average marginal effects (AMEs) for the explanatory variable (with Standard Errors in parentheses). Due to the categorical nature of our outcomes and explanatory variables, the AMEs are to be interpreted as the discrete effect of the independent variables (compared to the reference category). For categorical variables with more than two values, AMES show the difference (in percentage points) in predicted probabilities between categories relative to the reference category (for example, *no immediate family* vs *immediate family, low resources*). AMEs in this Table are presented as percentage points (divided by 100).

*** p < 0.001;

** p < 0.01;

* p < 0.05.

Source: English Longitudinal Study of Ageing (ELSA) waves 4 (2008/09) – 9 (2018/19).

**Table 4:** Trajectories of loneliness and the effects of time-varying predictors on trajectory shapes, by trajectory group.

	Group 1 *Stable low*	Class 2 *Increasing*	Class 3 *Decreasing*	Class 4 *Stable medium*	Class 5 *Stable high*
In paid work	0.215 (0.143)	0.127 (0.095)	0.022 (0.045)	0.014 (0.023)	0.025 (0.021)
Volunteered	−0.036 (0.245)	0.040 (0.129)	−0.051 (0.075)	−0.044 (0.023)	−0.026 (0.031)
Fair/poor SRH	0.533 (0.199) **	−0.055 (0.094)	0.072 (0.073)	0.140 (0.031) ***	−0.014 (0.031)
Disability	0.154 (0.187)	0.097 (0.095)	0.139 (0.070) *	0.081 (0.031) **	0.047 (0.028)
Depressed	1.542 (0.218) ***	0.867 (0.104) ***	0.567 (0.083) ***	0.448 (0.031) ***	0.305 (0.027) ***
Fair/Poor Vision or Hearing	0.504 (0.169) ***	0.165 (0.085) *	0.183 (0.059) ***	0.039 (0.028)	0.014 (0.026)
Sedentary Behaviour	0.023 (0.228)	0.116 (0.099)	0.212 (0.085) **	−0.019 (0.037)	0.005 (0.034)
No **partner**	0.523 (0.089) ***	0.476 (0.121) ***	0.361 (0.131) ***	0.303 (0.112) ***	0.204 (0.028) ***
Partner – Low resources	*Ref*	Ref	*Ref*	*Ref*	*Ref*
Partner – Medium resources	−0.519 (0.056)***	−0.647 (0.137) ***	−0.300 (0.106) ***	−0.527 (0.126) ***	−0.347 (0.034) ***
Partner – High resources	−1.146 (0.154) ***	−0.961 (0.165) ***	−0.627 (0.164) ***	−0.996 (0.148) ***	−0.508 (0.049) ***
No **children**	−0.006 (0.225)	0.138 (0.141)	0.036 (0.089)	0.045 (0.044)	0.041 (0.040)
Children – Low resources	*Ref*	*Ref*	*Ref*	*Ref*	Ref
Children – Medium resources	−0.553 (0.213) **	−0.171 (0.084) *	−0.327 (0.066) ***	−0.129 (0.031) ***	−0.089 (0.029) ***
Children – High resources	−0.612 (0.235) **	−0.210 (0.095)*	−0.554 (0.096) ***	−0.291 (0.041) ***	−0.143 (0.037) ***
No **immediate family**	−0.103 (0.296)	−0.037 (0.149)	0.051 (0.126)	0.023 (0.050)	−0.028 (0.046)
Immediate Family – Low resources	*Ref*	*Ref*	*Ref*	*Ref*	*Ref*
Immediate Family – Medium resources	0.017 (0.190)	0.082 (0.107)	−0.116 (0.066)	−0.032 (0.029)	−0.038 (0.029)
Immediate Family – High resources	−0.217 (0.205)	−0.107 (0.109)	−0.181 (0.069) **	−0.165 (0.033) ***	−0.071 (0.031) *
No **friends**	0.348 (0.271)	0.554 (0.132) ***	0.239 (0.102) **	0.121 (0.052) *	0.087 (0.037) *
Friends – Low resources	*Ref*	*Ref*	*Ref*	*Ref*	Ref
Friends – Medium resources	−0.435 (0.191) *	−0.008 (0.100)	−0.101 (0.066)	−0.096 (0.030) ***	−0.057 (0.028) *
Friends – High resources	−0.979 (0.243) ***	−0.161 (0.077) *	−0.414 (0.077) ***	−0.195 (0.034) ***	−0.147 (0.033) ***

Notes: Estimates for time-varying covariates represent the shift in the average loneliness score per unit change in exposure variable (or per each category compared to the reference). The effects of time-varying predictors are estimated for each trajectory group, and thus, the effects could differ in each trajectory group. All models are adjusted for time-stable covariates gender, age groups, education, and wealth at baseline.

Standard errors in parentheses.

*** p < 0.001;

** p < 0.01;

* p < 0.05.

Source: English Longitudinal Study of Ageing (ELSA) waves 4 (2008/09) – 9 (2018/19).
