# Supplementary material for: Trajectories of loneliness in later life – Evidence from a 10-year English panel study
Source: Soc Sci Med. Author manuscript; Available in PMC 2025 Jul 16. (PMC12264031; doi:10.1016/j.socscimed.2025.117703)
Supplement: Appendix A [file NIHMS2061018-supplement-Appendix_A.docx]

**Supplementary Table 1. Comparison of goodness of fit criteria for group-based trajectory modelling models of longitudinal loneliness**

| **N classes** | **AIC** | **BIC** | **cBIC** | **Entropy** | **Class membership (%)** |
| --- | --- | --- | --- | --- | --- |
| 1 | -41847.4 | -41855.6 | -41853.8 | 1 |  |
| 2 | -32470.5 | -32491.1 | -32486.7 | 0.897 | 59.4; 40.6 |
| 3 | -31243.2 | -31276.0 | -31269.0 | 0.824 | 43.5; 34.9; 21.6 |
| 4 | -31037.9 | -31083.1 | -31073.5 | 0.755 | 37.2; 28.5; 25.8; 8.5 |
| **5** | **-30837.3** | **-30894.8** | **-30882.6** | **0.741** | **37.4, 25.8, 20.4, 8.5, 7.9** |
| 6 | -30801.6 | -30871.3 | -30856.5 | 0.716 | 36.0, 22.1, 21.8, 8.7, 6.4, 5.0 |
| 7 | -30777.7 | -30859.8 | -30842.4 | 0.671 | 28.1; 20.7; 18.0; 15.3; 8.3; 4.8; 4.8 |

Source: ELSA waves 4-9 (N=4740). Notes: AIC=Akaike Information Criterion; BIC=Bayesian Information Criterion; c-BIC=sample size corrected BIC

**Supplementary Table 2. Comparison of goodness of fit criteria for 5 groups the establish the optimal shape of the loneliness trajectories**

| **Polynomic function** | **cBIC** | **Entropy** | **APPA** | | | | | **OCC** | | | | |
| --- | --- | --- | --- | --- | --- | --- | --- | --- | --- | --- | --- | --- |
|  |  |  | **G1** | **G2** | **G3** | **G4** | **G5** | **G1** | **G2** | **G3** | **G4** | **G5** |
| 33333 | -30908.2 | 0.742 | 0.912 | 0.778 | 0.751 | 0.818 | 0.867 | 18.8 | 13.3 | 29.6 | 13.7 | 60.8 |
| 33332 | -30929.1 | 0.718 | 0.894 | 0.746 | 0.761 | 0.825 | 0.785 | 19.7 | 10.6 | 30.2 | 13.4 | 48.2 |
| 33331 | -30899.8 | 0.742 | 0.912 | 0.778 | 0.753 | 0.818 | 0.864 | 18.8 | 13.3 | 29.9 | 13.7 | 58.8 |
| 33321 | -30896.5 | 0.742 | 0.912 | 0.778 | 0.756 | 0.816 | 0.869 | 18.8 | 13.2 | 30.5 | 13.5 | 61.5 |
| 23321 | -30892.6 | 0.742 | 0.912 | 0.778 | 0.757 | 0.816 | 0.869 | 18.8 | 13.3 | 30.6 | 13.5 | 61.8 |
| 23311 | -30889.7 | 0.742 | 0.912 | 0.779 | 0.759 | 0.816 | 0.869 | 18.9 | 13.2 | 31.1 | 13.5 | 61.8 |
| **23310** | **-30885.6** | **0.742** | **0.912** | **0.778** | **0.756** | **0.817** | **0.866** | **18.9** | **13.2** | **30.6** | **13.7** | **60.8** |
| 22311 | -30887.9 | 0.742 | 0.911 | 0.780 | 0.753 | 0.818 | 0.868 | 18.6 | 13.4 | 30.3 | 13.7 | 61.1 |

Source: ELSA waves 4-9 (N=4740). Notes: 3=Cubic trajectory; 2=Quadratic Trajectory; 1=Linear Trajectory; 0=Intercept. BIC=Bayesian Information Criteria; c-BIC=sample size corrected BIC; APPA= Average Posterior Probability of Assignments; OCC=Odds of correct classification. The model that best fits the data is in bold – this the model has the lowest c- BIC, all APPA>70% for each class, and OCC>5.0 for each class.

**Supplementary Table 3. Estimates of unconditional group-based trajectory models**

| **Group** | **Parameter** | **Estimate** | **SE** | **P value** |
| --- | --- | --- | --- | --- |
| 1 | Intercept | 1.498 | 1.01 | 0.138 |
|  | Linear | -1.387 | 0.34 | <0.001 |
|  | Quadratic | 0.097 | 0.03 | <0.001 |
| 2 | Intercept | 10.417 | 4.373 | 0.017 |
|  | Linear | -6.149 | 2.09 | 0.003 |
|  | Quadratic | 0.995 | 0.31 | 0.001 |
|  | Cubic | -0.048 | 0.01 | 0.001 |
| 3 | Intercept | -2.888 | 1.58 | 0.068 |
|  | Linear | 1.724 | 0.81 | 0.034 |
|  | Quadratic | -0.311 | 0.13 | 0.020 |
|  | Cubic | 0.016 | 0.01 | 0.028 |
| 4 | Intercept | 0.701 | 0.05 | <0.001 |
|  | Linear | -0.013 | 0.01 | 0.059 |
| 5 | Intercept | 1.269 | 0.02 | <0.001 |

Source: ELSA waves 4-9 (N=4740). Values obtained from 5 groups with 2 cubic trajectories (Groups 2 and 3); one quadratic (Group 1); one linear (Group 4) and one intercept only (Group 5).

**Supplementary Table 4 - Average R-UCLA loneliness scores (and percentage of respondents who reported a score of zero) over time across classes**

|  | **Wave 4** | **Wave 5** | **Wave 6** | **Wave 7** | **Wave 8** | **Wave 9** |
| --- | --- | --- | --- | --- | --- | --- |
| **Class 1 –** *stable low loneliness* | 0.07 (93%) | 0.04 (96%) | 0.04 (96%) | 0.03 (97%) | 0.04 (96%) | 0.04 (96%) |
| **Class 2 –** *increasing loneliness* | 0.16 (86%) | 0.17 (85%) | 0.26 (78%) | 0.57 (59%) | 1.39 (25%) | 1.72 (13%) |
| **Class 3 –** *decreasing loneliness* | 1.13 (32%) | 0.89 (43%) | 0.79 (47%) | 0.46 (64%) | 0.32 (72%) | 0.28 (75%) |
| **Class 4 –** *stable medium loneliness* | 1.90 (16%) | 1.89 (15%) | 2.02 (10%) | 1.86 (14%) | 1.81 (14%) | 1.81 (17%) |
| **Class 5 –** *stable high loneliness* | 3.77 (3%) | 3.76 (2%) | 3.82 (1%) | 3.71 (1%) | 3.91 (1%) | 3.71 (1%) |

Source: English Longitudinal Study of Ageing (ELSA) waves 4 (2008/09) – 9 (2018/19)

**Supplementary Table 5. Descriptive statistics of the time-varying covariates by loneliness trajectories**

|  |  | **Group 1**  *Stable low* | **Class 2**  *Increasing* | **Class 3**  *Decreasing* | **Class 4**  *Stable medium* | **Class 5**  S*table high* | **Tot** |
| --- | --- | --- | --- | --- | --- | --- | --- |
| **Health** | | | | | | | |
| Poor Self-Rated Health | Wave 4 | 10.3 | 16.8 | 16.0 | 21.4 | 39.9 | 17.4 |
|  | Wave 5 | 10.4 | 17.2 | 16.0 | 20.6 | 39.0 | 17.1 |
|  | Wave 6 | 11.9 | 22.6 | 18.9 | 26.1 | 42.5 | 20.4 |
|  | Wave 7 | 12.3 | 22.4 | 18.6 | 25.6 | 43.0 | 20.4 |
|  | Wave 8 | 15.0 | 25.3 | 18.7 | 28.1 | 46.8 | 22.7 |
|  | Wave 9 | 16.5 | 30.3 | 21.3 | 33.5 | 44.8 | 25.4 |
| Disability | Wave 4 | 11.8 | 18.2 | 16.1 | 23.5 | 37.1 | 18.4 |
|  | Wave 5 | 11.9 | 19.9 | 16.7 | 22.8 | 35.8 | 18.3 |
|  | Wave 6 | 12.5 | 19.8 | 17.9 | 24.6 | 41.9 | 19.8 |
|  | Wave 7 | 12.9 | 20.9 | 16.4 | 25.9 | 41.6 | 20.0 |
|  | Wave 8 | 15.4 | 24.6 | 18.8 | 27.9 | 45.5 | 22.6 |
|  | Wave 9 | 17.8 | 32.2 | 21.4 | 32.0 | 48.0 | 25.9 |
| Depressed | Wave 4 | 3.2 | 4.0 | 7.9 | 14.2 | 42.3 | 10.4 |
|  | Wave 5 | 5.9 | 9.0 | 11.8 | 26.0 | 55.3 | 16.6 |
|  | Wave 6 | 2.2 | 5.2 | 5.5 | 14.3 | 42.0 | 9.6 |
|  | Wave 7 | 1.6 | 8.5 | 3.4 | 13.8 | 43.2 | 9.2 |
|  | Wave 8 | 1.8 | 8.1 | 4.3 | 14.3 | 42.4 | 9.5 |
|  | Wave 9 | 2.7 | 11.3 | 4.4 | 14.8 | 43.8 | 10.3 |
| Fair/Poor Vision or Hearing | Wave 4 | 18.9 | 23.8 | 22.5 | 23.9 | 32.1 | 22.4 |
|  | Wave 5 | 18.6 | 24.3 | 23.0 | 27.2 | 33.8 | 23.4 |
|  | Wave 6 | 20.5 | 27.7 | 25.3 | 30.1 | 34.8 | 25.7 |
|  | Wave 7 | 21.1 | 27.3 | 23.7 | 31.5 | 34.3 | 25.9 |
|  | Wave 8 | 24.3 | 33.2 | 27.5 | 31.9 | 38.1 | 28.8 |
|  | Wave 9 | 24.3 | 37.7 | 28.3 | 36.1 | 41.5 | 30.7 |
| Sedentary Behaviour | Wave 4 | 6.5 | 9.9 | 8.5 | 9.9 | 18.2 | 9.0 |
|  | Wave 5 | 6.6 | 11.4 | 7.3 | 10.8 | 16.6 | 9.0 |
|  | Wave 6 | 7.7 | 11.4 | 9.7 | 12.8 | 18.4 | 10.6 |
|  | Wave 7 | 7.1 | 11.9 | 10.3 | 15.1 | 23.1 | 11.5 |
|  | Wave 8 | 9.8 | 18.8 | 10.7 | 18.7 | 29.1 | 14.6 |
|  | Wave 9 | 12.4 | 23.8 | 14.8 | 22.4 | 32.6 | 18.1 |
| **Social Relationships** | | | | | | | |
| No partner | Wave 4 | 11.7 | 11.4 | 23.1 | 31.3 | 48.4 | 22.2 |
|  | Wave 5 | 11.2 | 12.9 | 23.5 | 33.3 | 49.9 | 22.6 |
|  | Wave 6 | 11.3 | 13.5 | 24.1 | 35.1 | 51.8 | 23.5 |
|  | Wave 7 | 11.8 | 16.9 | 24.7 | 36.9 | 57.1 | 25.1 |
|  | Wave 8 | 12.6 | 26.1 | 25.1 | 39.3 | 59.3 | 27.0 |
|  | Wave 9 | 14.0 | 35.0 | 26.8 | 40.7 | 59.7 | 29.0 |
| Average resources partner^*^ | Wave 4 | 18.35 | 17.50 | 16.62 | 15.70 | 12.83 | 17.01 |
|  | Wave 5 | 18.14 | 17.19 | 16.56 | 15.34 | 12.24 | 16.81 |
|  | Wave 6 | 18.26 | 17.28 | 16.68 | 15.37 | 11.97 | 16.89 |
|  | Wave 7 | 18.36 | 17.05 | 17.07 | 15.67 | 12.12 | 17.10 |
|  | Wave 8 | 18.12 | 16.52 | 16.97 | 15.46 | 11.84 | 16.89 |
|  | Wave 9 | 18.11 | 15.90 | 17.10 | 15.39 | 12.04 | 16.86 |
| No children | Wave 4 | 12.9 | 9.7 | 13.1 | 14.7 | 16.7 | 13.5 |
|  | Wave 5 | 13.1 | 10.5 | 13.2 | 14.8 | 16.9 | 13.6 |
|  | Wave 6 | 13.2 | 11.1 | 13.3 | 14.3 | 15.7 | 13.6 |
|  | Wave 7 | 12.9 | 11.5 | 13.8 | 14.7 | 17.5 | 13.8 |
|  | Wave 8 | 12.8 | 10.4 | 13.5 | 15.4 | 17.0 | 13.8 |
|  | Wave 9 | 13.2 | 10.6 | 13.3 | 15.3 | 16.5 | 13.8 |

|  |  | **Group 1**  *Stable low* | **Class 2**  *Increasing* | **Class 3**  *Decreasing* | **Class 4**  *Stable medium* | **Class 5**  S*table high* | **Tot** |  |
| --- | --- | --- | --- | --- | --- | --- | --- | --- |
| Average resources children^*^ | Wave 4 | 9.36 | 9.20 | 8.72 | 8.39 | 7.82 | 8.84 |  |
|  | Wave 5 | 9.42 | 9.14 | 8.91 | 8.43 | 7.56 | 8.89 |  |
|  | Wave 6 | 9.50 | 9.36 | 9.03 | 8.47 | 8.73 | 8.99 |  |
|  | Wave 7 | 9.49 | 9.23 | 9.06 | 8.57 | 7.80 | 9.01 |  |
|  | Wave 8 | 9.71 | 9.26 | 9.21 | 8.62 | 7.75 | 9.14 |  |
|  | Wave 9 | 9.77 | 9.28 | 9.35 | 8.84 | 7.84 | 9.25 |  |
| No immediate family | Wave 4 | 6.9 | 4.9 | 5.0 | 7.3 | 6.0 | 6.4 |  |
|  | Wave 5 | 6.7 | 5.4 | 5.1 | 6.5 | 6.9 | 6.2 |  |
|  | Wave 6 | 7.1 | 5.9 | 5.2 | 6.1 | 8.1 | 6.4 |  |
|  | Wave 7 | 7.3 | 6.3 | 6.6 | 8.6 | 8.2 | 7.5 |  |
|  | Wave 8 | 8.1 | 7.6 | 5.9 | 7.9 | 10.0 | 7.7 |  |
|  | Wave 9 | 8.5 | 8.9 | 9.2 | 9.0 | 11.8 | 9.1 |  |
| Average resources family^*^ | Wave 4 | 9.12 | 8.92 | 8.41 | 8.28 | 7.47 | 8.60 |  |
|  | Wave 5 | 9.40 | 9.34 | 8.72 | 8.59 | 7.88 | 8.92 |  |
|  | Wave 6 | 9.41 | 9.20 | 8.81 | 8.59 | 8.06 | 8.95 |  |
|  | Wave 7 | 9.56 | 9.36 | 9.04 | 8.72 | 7.93 | 9.09 |  |
|  | Wave 8 | 9.24 | 8.91 | 8.63 | 8.24 | 7.32 | 8.67 |  |
|  | Wave 9 | 9.12 | 8.89 | 8.94 | 8.24 | 7.66 | 8.72 |  |
| No friends | Wave 4 | 2.1 | 3.5 | 3.7 | 4.4 | 8.6 | 3.7 |  |
|  | Wave 5 | 2.7 | 3.4 | 5.1 | 5.0 | 9.9 | 4.4 |  |
|  | Wave 6 | 3.3 | 3.9 | 5.0 | 6.8 | 10.6 | 5.2 |  |
|  | Wave 7 | 3.5 | 6.4 | 4.6 | 6.3 | 12.6 | 5.4 |  |
|  | Wave 8 | 3.7 | 7.3 | 5.1 | 7.3 | 11.6 | 5.9 |  |
|  | Wave 9 | 3.6 | 7.1 | 4.7 | 6.8 | 13.5 | 5.7 |  |
| Average resources friends^*^ | Wave 4 | 11.62 | 10.99 | 10.87 | 10.69 | 10.01 | 11.05 |  |
|  | Wave 5 | 11.60 | 10.91 | 10.89 | 10.49 | 9.77 | 10.98 |  |
|  | Wave 6 | 11.57 | 10.98 | 10.95 | 10.68 | 9.78 | 11.03 |  |
|  | Wave 7 | 11.48 | 10.98 | 10.95 | 10.43 | 9.72 | 10.93 |  |
|  | Wave 8 | 11.48 | 10.77 | 10.93 | 10.46 | 9.42 | 10.89 | |
|  | Wave 9 | 11.25 | 10.67 | 10.86 | 10.19 | 9.35 | 10.71 | |
| **Socioeconomic characteristics** | | | | | | | |  |
| In paid work | Wave 4 | 48.1 | 39.0 | 49.4 | 39.7 | 36.8 | 44.5 | |
|  | Wave 5 | 39.0 | 32.6 | 41.0 | 32.7 | 29.1 | 36.5 | |
|  | Wave 6 | 32.3 | 25.4 | 34.4 | 25.5 | 23.9 | 29.7 | |
|  | Wave 7 | 25.9 | 19.5 | 29.1 | 21.2 | 19.7 | 24.3 | |
|  | Wave 8 | 19.1 | 15.8 | 19.7 | 16.5 | 14.2 | 17.9 | |
|  | Wave 9 | 15.6 | 10.4 | 16.2 | 13.0 | 12.5 | 14.4 | |
| Volunteered | Wave 4 | 19.8 | 17.1 | 17.2 | 18.9 | 12.9 | 18.2 | |
|  | Wave 5 | 22.0 | 17.2 | 18.7 | 18.9 | 13.8 | 19.5 | |
|  | Wave 6 | 22.4 | 20.7 | 21.4 | 18.9 | 15.4 | 20.5 | |
|  | Wave 7 | 23.7 | 20.7 | 19.1 | 17.9 | 14.2 | 20.1 | |
|  | Wave 8 | 24.0 | 17.7 | 20.9 | 18.0 | 17.0 | 20.7 | |
|  | Wave 9 | 24.5 | 17.4 | 21.8 | 18.4 | 14.4 | 20.9 | |
| Lowest Wealth Tertile | Wave 4 | 25.6 | 33.1 | 31.8 | 36.7 | 53.7 | 32.7 | |
|  | Wave 5 | 24.8 | 33.7 | 30.3 | 37.9 | 50.8 | 32.3 | |
|  | Wave 6 | 24.3 | 31.0 | 30.8 | 39.3 | 52.0 | 32.4 | |
|  | Wave 7 | 25.4 | 32.1 | 30.3 | 38.0 | 50.8 | 33.2 | |
|  | Wave 8 | 25.7 | 31.3 | 29.4 | 37.8 | 50.8 | 32.2 | |
|  | Wave 9 | 26.1 | 33.2 | 30.3 | 38.2 | 54.7 | 33.1 | |

Source: English Longitudinal Study of Ageing (ELSA) waves 4 (2008/09) – 9 (2018/19).
Note: * Mean scores were calculated among respondents who reported having social relationships in the different domains considered (partner, children, immediate family, friends).
